# Depression and HIV Risk Behaviors among Female Sex Workers in Guangdong, China: A Multicenter Cross-Sectional Study

**DOI:** 10.1155/2016/6986173

**Published:** 2016-12-20

**Authors:** Hongcheng Shen, Huachun Zou, Shujie Huang, Fengying Liu, Peizhen Zhao, Lei Chen, Ye Zhang, Xiaomin Luo, Weiming Tang, Heping Zheng, Bin Yang

**Affiliations:** ^1^Guangdong Provincial Center for Skin Disease and STI Control, Guangzhou, Guangdong 510091, China; ^2^School of Public Health, Sun Yat-sen University, Guangzhou, Guangdong 510085, China; ^3^Kirby Institute, The University of New South Wales, Sydney, NSW 2052, Australia; ^4^Guangdong Pharmaceutical University, Guangzhou, Guangdong 511430, China; ^5^University of North Carolina Project-China, Guangzhou, Guangdong, 510091, China

## Abstract

*Background.* Our study aimed to assess the burden of depression and evaluate factors associated with depression and status of HIV risk behaviors among female sex workers (FSWs) in Guangdong, China.* Method.* We recruited FSWs from massage parlors, saunas, restaurants, hotels, hair salons, and streets in Guangdong, China, in 2014. Information on demographic characteristics, HIV testing history, and sexual behaviors was collected using a questionnaire. A blood sample was collected to test for HIV, syphilis, and HCV. A participant was defined as being depressed if she obtained 6 points or above using the 12-item General Health Questionnaire.* Results.* Among the 653 participants, 41.7% were 21–30 years old and 43.6% married. Overall, 52.4% were found to be depressed. FSWs who had correct syphilis related knowledge [aOR = 1.45; 95% CI: 1.04–2.03] and had primary sex partner (1.63, 1.14–2.33) were more likely to be depressed. FSWs who did not use a condom during their last sex with the primary sex partner were less likely to be depressed (0.47, 0.31–0.71).* Conclusion.* Our study observed high level of depression and HIV risk behaviors among Chinese FSWs. Future interventions should integrate mental health services in comprehensive interventions to prevent depression among Chinese FSWs.

## 1. Introduction

Female sex workers (FSWs) are disproportionately affected by HIV and other sexually transmitted diseases (STDs). The pooled HIV prevalence in FSWs in 2012 was 11.8% based on data from 50 countries, including China [1]. It was estimated that there were 2–20 million FSWs in China [2–5]. FSWs are at high risk of HIV acquisition and are a bridge for the transmission of HIV/STDs to the general population [6]. A recent meta-analysis revealed that during 2000–2011 the high prevalence of STDs among FSWs became a severe public health issue in China [7].

Besides HIV/STDs, FSWs face severe stigma, sexual violence, and social discrimination [1]. As a marginalized population, they also tend to be socially isolated. FSWs are often portrayed as a symbol of social evil in China [8]. The discrimination against them became even harsher in recent years, rendering them subject to depression [9]. Existing literature on Chinese FSWs usually focused on the burden of HIV/STDs among them; however, mental health problems among this population had been neglected [10].

Globally, literature has revealed that HIV/STDs risk factors such as work-related violence [11], unwanted pregnancy [12], and condomless sex [13] were associated with mental health problems, and mental health problems in turn may increase the spread of HIV/STDs among FSWs since FSWs with high level of depression symptoms were less likely to use condom consistently or properly [8]. FSWs with mental health problem are more likely to have suicide ideation and attempt [11–14]. Limited pilot studies have indicated that partner violence, inconsistent condom use, and nonuse of prevention service were associated with mental health problems among FSWs in China [15, 16]. Until now, very few studies had assessed depression among FSWs in China. Our study aimed to assess the burden of and factors associated with depression and status of HIV risk behaviors among FSWs in Guangdong, China.

## 2. Methods

### 2.1. Study Sample

In this multicenter, cross-sectional study, geographical mapping was used to identify the commercial sex entertainment venues. Participants in this study were recruited from massage parlors, saunas, restaurants, hotels, hair salons, and streets in three cities (Zhuhai, Nanshan District in Shenzhen, and Yingde City in Qingyuan) in Guangdong, southern China, between June and November in 2014. These three sites were chosen according to their income level: Nanshan District, a high-income area with an incidence of newly reported syphilis of 65 per 100000 person-years in 2013; Zhuhai, a medium-income area with an incidence of newly reported syphilis of 55 per 100000 person-years in 2013; and Yingde, a low-income area with an incidence of newly reported syphilis of 76 per 100000 person-years in 2013 (surveillance data not published). Convenient sampling method was used to recruit eligible participants. Outreach strategies to contact FSWs and collect data were as follows: first, trained investigators contacted the owners or managers of each establishment for their permission to conduct the survey on their premises. Second, investigators invited FSWs in participating venues to participate in our survey. Third, investigators introduced the survey to FSWs in detail and asked them to provide informed consent before completing it. Eligible participants must meet the following inclusion criteria: (1) female gender by birth, (2) aged 18 years or older, and (3) having sold sex for money or goods in the last year.

### 2.2. Data Collection

#### 2.2.1. Demographic and Socioeconomic Measures

Trained investigators conducted face-to-face interviews using a structured questionnaire. Demographic and socioeconomic information, including age, education level, marital status, living status, monthly income, and residency, was collected from each participant. Age (in years) was measured as a continuous variable and was further categorized into different age groups (<20; 21–30; 31–40; 41 and above). Education was measured by the highest level of education obtained and categorized into primary school and below, middle school, and senior high school and above. Marital status was categorized into unmarried, married, and divorced or widowed. Living status was categorized into living alone, living with partners, and others. Monthly income was categorized into three groups (in USD): 450 and lower, 451–749, and 750 and higher. Residence was categorized into study city, another city in Guangdong province, and other provinces.

#### 2.2.2. Behavior Characteristics

Sexual behaviors variable included experience of HIV/STDs services in the past year (including education promotion, HIV/STDs counseling and testing, and condom promotion), syphilis related knowledge, condom use during the last sex with a client, frequency of condom use in the last month with clients, condom use during the last sex with primary sex partner, frequency of condom use in the last month with primary sex partners, STD symptoms in the past year, and drug use. Participants received any kind of HIV/STD health service in the last year were considered to have experienced HIV/STD service. Participants providing correct answers to at least 6 of the 8 syphilis related knowledge questions were considered to have correct knowledge regarding syphilis transmission and prevention. Participants having any related HIV/STD symptom in last year were considered to have HIV/STD symptom. Participants having ever taken any kind of drug were considered to be drug user.

#### 2.2.3. Depression Assessment

The depression status of the participants was assessed using the validated 12-item General Health Questionnaire (GHQ-12) [17]. The Chinese version of the questionnaire had been validated in the context of China [18]. The GHQ-12 is a screening tool which was used to screen the severity of depression experienced by an individual within the past few weeks. Each item on the scale has four answers including “better than usual,” “same as usual,” “less than usual,” and “much less than usual.” For the purpose of this study, the scoring method (0-0-1-1) was chosen. Total scores of each participant were summed up using all the items score ranging from 0 to 12. Due to the various thresholds of the GHQ-12, the mean GHQ score for the participants was suggested as a rough indicator for the best cut-off point [19]. Therefore, based on the mean GHQ-12 score for this sample, 6 as the cut-off point was used to determine participants' level of depression.

#### 2.2.4. Serologic Measures

Trained investigators collected 5 ml of venous blood to test for HIV, syphilis, and HCV. HIV antibodies were screened using an enzyme-linked immune sorbent assay (ELISA; Zhuhai LiZhu Co., Ltd.). Positive samples were doubled-checked by another enzyme-linked immune sorbent assay (ELISA; Beijing Wantai Biological Pharmacy Enterprise Co., Ltd.). Samples positive by both ELISA tests were further confirmed by Western blot. Syphilis antibodies were screened using an enzyme-linked immune sorbent assay (ELISA; Zhuhai LiZhu Co., Ltd.). Positive samples were further confirmed by Toluidine Red Unheated Serum Test (TRUST; Shanghai RongSheng). Syphilis positivity was deemed as “current” when both ELISA and TRUST assays were positive. HCV antibodies were screened using an enzyme-linked immune sorbent assay (ELISA; Beijing Wantai Biological Pharmacy Enterprise Co., Ltd.). Positive samples were double-checked by another enzyme-linked immune sorbent assay (ELISA; Zhuhai LiZhu Co., Ltd.). Samples positive by both ELISA tests were defined as HCV positive.

### 2.3. Statistical Analysis

Data were double-entered with logic checks, using EpiData 3.0. Identified errors were corrected by communicating with the local chronic diseases prevention and control hospitals and rechecking the original questionnaires. Descriptive analyses were conducted to determine the distribution of the demographic factors, behaviors, HIV and syphilis prevalence, and other related information for both participants who were identified as depressed and not. Univariate logistic regression analyses with odds ratio (OR) and corresponding 95% confidence interval (CI) were used to identify predictors of depression among the participants. To control for potential confounders, multivariate logistic regressions were further performed to determine adjusted ORs (aOR) and corresponding 95% CIs. Variables with a *p* value <0.1 was entered in the multivariate logistic regressions. Age, education level, marital status, and monthly income were also included in the multivariate logistic regression model. SAS version 9.2 (SAS Ins., Cary, NC, USA) was used for all statistical analyses.

### 2.4. Ethics Approval

Signed informed consent was obtained from each of the participants prior to interview and blood collection. Each of the participants was free to withdraw from this survey at any time after recruitment without any consequence. The study protocol was reviewed and approved by the Ethics Committee of the Guangdong Provincial Center for Skin Disease and STI Control in Guangzhou.

## 3. Results

### 3.1. Demographic Characteristics

A total of 653 participants were recruited from the three cities (120 from Shenzhen, 300 from Zhuhai, and 233 from Qingyuan). More than two-fifths (272/653, 41.7%) of the participants were 21–30 years old and 43.6% (281/645) were married. More than half (375/653, 57.4%) had an education of middle school. The majority of participants had a monthly income less than USD750 (589/653, 90.2%). Most were migrants from other provinces (458/646, 70.9%). More than four-fifths (415/514, 80.7%) had received STDs related service in the last year, and approximately two-fifths (261/653, 40.0%) had correct syphilis related knowledge. In addition, 33.5% (219/653) of the participants had ever experienced STDs related symptoms in the past year (Table 1).

### 3.2. Behavioral Characteristics

More than three-fifths (434/653, 66.5%) of the participants self-reported that they currently had primary sex partners. Just over one-fifth (133/648, 20.5%) of the participants did not use a condom during their last sex with a client, and 43.9% (190/433) did not use a condom during their last sex with their primary sex partner. Among the participants, 36.6% (239/653) engaged in condomless sex with clients in the last month, and 74.1% (484/653) engaged in condomless sex with primary sex partner in the last month. Only 1.1% (7/653) of the participants were drug users (Table 1).

### 3.3. HIV, Syphilis, and HCV

HIV, syphilis, and HCV were detected in 0.6% (4/653), 5.1% (33/653), and 2.0% (13/653) of participants. The HIV prevalence among non-depression group and depression group were 0.3% (95% CI: 0.0–1.0%) and 0.9% (0.0–1.9%), respectively. Syphilis prevalence for the two groups was 6.4% (3.7–9.2%) and 3.8% (1.8–5.8%), respectively. HCV prevalence was 1.6% (0.2–3.0%) and 2.3% (0.7–3.9%) among the two groups, respectively (Table 1).

### 3.4. Assessment of Depression

Table 2 shows the mean score for each item of GHQ-12 among the participants. Over half (52.4%) of FSWs obtained 6 points or higher. Item 10 “been losing confidence in yourself” had the highest mean score of 0.91, while item 7 “been able to enjoy your normal day-to-day activities” had the lowest mean score of 0.04.

The results of univariate analysis indicated that compared to participants with a monthly income USD450 and lower, those with a monthly income USD750 and higher were more likely to be depressed (OR = 1.95, 1.11–3.45). Compared to participants from study cities, participants from other cities in Guangdong province were more likely to be depressed (OR = 2.03, 1.06–3.89). After adjusting for age, education, marital status, and monthly income, results from multivariate analysis indicated participants who had syphilis related knowledge (aOR = 1.45, 1.04, 2.03) were more likely to be depressed compared to those who did not. The study results further indicated that FSWs who had primary sex partner were more likely to be depressed (aOR = 1.63, 1.14–2.33). We also found that FSWs who did not use a condom during their last sex with primary sex partner (aOR = 0.47, 0.31–0.71) were less likely to be depressed. Participants tested positive for HIV or syphilis; however, they did not have statistically significantly higher rate of depression (Table 3).

## 4. Discussion

The current study was the first one in Guangdong to report on the severity and associated factors of depression among FSWs. The results revealed that depression and HIV risk behaviors were high among FSWs in Guangdong.

In our study, participants with syphilis related knowledge were more likely to be depressed. One potential reason for this phenomenon is that correct knowledge on syphilis may make the participants more anxious about their health when they engage in commercial sex, and, in turn, lead to the problem of depression. FSWs may expose to risk behavior or have previous or current HIV/STDs related symptoms. As a result they may be more alert to and worried about health issues, which subject them to depression. This result indicates that health care service should be strengthened to avoid depression among FSWs on a timely basis.

Our result also indicated that participants with primary sex partner were more vulnerable to depression. This finding was consistent with another study conducted in southwest China, which reported that FSWs were more likely to be victims of violence from regular partner [20]. Another study focused on FSWs in China also observed that primary violence was a constant source of stress and more detrimental effects such as alcohol intoxication and suicidal behavior [15]. In China, partners of FSWs may be financially reliant on FSWs [21]. Future intervention program should involve primary sex partner of FSWs. We also identified that FSWs who did not use a condom during their last sex with primary sex partner were less likely to be depressed. FSWs may have barrier to use condoms with their primary sex partner as it may affect the intimacy of the relationship or as an implication of mistrust [22]. They may also see condom use as a way of distinguishing work-related sexual relationship from personal relationship [21]. FSWs tend not to use a condom during sexual intercourse with their primary sex partners. Inorder to maintain trust in their primary sexual relationship, FSWs may choose not to use a condom with their primary sex partners [23, 24]. Condom promotion should target both FSWs and their primary sex partners.

Our data indicated that an extremely high proportion (52.4%) of the participants has the potential to develop or experience depression disorder which was higher than that reported in studies from Sydney, Australia (33.3%, female street-based sex workers) [11], Guangxi, China (39%, female injection drug user who are sex workers) [14], Tijuana and Ciudad Juarez, Mexico (11.8%, female sex workers who use drugs) [21], but lower than studies from Nepal (82.4%, female sex workers) [13]. Previous studies indicated that FSWs with mental health problems were more likely to have suicide ideation and attempt [11–14] and FSWs with high level of depression symptoms were less likely to use condom consistently or properly [8] which made them at high risk of HIV/STDs infection. We searched in PubMed and the Chinese National Knowledge Infrastructure (CNKI) databases using the key words “female sex workers” and “depression” but did not find any study that reported on intervention of depression among FSWs in either China or other countries. Depression disorder has now been recognized in the literature but poorly addressed in public health programs; therefore more attention needs to be paid to the mental health wellbeing of FSWs in China [9].

Our study had several limitations. Due to the nature of convenience sampling, our sample may not be representative of FSWs in other parts of China. As the data were collected through face-to-face interviews, our study may be subject to social desirability bias, which might have led to exposure or confounder misclassifications. Even though we adjusted for age, education, marital status, and monthly income in the multivariate analysis models, our study might still have residual confounding due to the remaining unknown or unadjusted confounders. We acknowledge the potential differences in various depression assessment scales. When comparing proportion of depression among different studies, the difference we detect may vary from the true difference if a universal scale was used in those studies.

In conclusion, Chinese FSWs were suffering from extremely high level of depression. Results of our study indicated prevalent depression was relatively high among Chinese FSWs, which may contribute to the increase in HIV/STD transmission among FSWs and their clients/sexual partners. Future intervention should strengthen symptom management after education promotion, involve primary sex partners of FSWs, and integrate mental health service in comprehensive intervention to prevent depression among Chinese FSWs.

## Figures and Tables

**Table 1:** Distribution of socio-demographics, sexual behavior and HIV/syphilis prevalence among female sex workers in Guangdong, China (*N* = 653).

Item	Non-depression (*n* = 311)			Depression (*n* = 342)			Total (*n* = 653)	
	*n*	%	95% CI	*n*	%	95% CI	*n*	%
Age (Year)								
≤20	38	12.2	8.6, 15.9	53	15.5	11.6, 19.4	91	13.9
21–30	139	44.7	39.1, 50.3	133	38.9	33.7, 44.1	272	41.7
31–40	77	24.8	19.9, 29.6	93	27.2	22.5, 31.9	170	26.0
41 and above	57	18.3	14.0, 22.7	63	18.4	14.3, 22.6	120	18.4
Education								
Primary school and below	66	21.2	16.7, 25.8	87	25.4	20.8, 30.1	153	23.4
Middle school	188	60.5	55.0, 65.9	187	54.7	49.4, 60.0	375	57.4
Senior high school and above	57	18.3	14.0, 22.7	68	19.9	15.6, 24.1	125	19.1
Marital Status								
Unmarried	121	39.8	34.3, 45.3	152	44.6	39.3, 49.9	273	42.3
Married	137	45.1	39.4, 50.7	144	42.2	37.0, 47.5	281	43.6
Others	46	15.1	11.1, 19.2	45	13.2	9.6, 16.8	91	14.1
Living Status								
Alone	162	52.4	46.8, 58.0	165	48.5	43.2, 53.9	327	50.4
With partner	72	23.3	18.6, 28.0	88	25.9	21.2, 30.6	160	24.7
Others	75	24.3	19.5, 29.1	87	25.6	20.9, 30.3	162	25.0
Monthly income								
≤450	144	46.3	40.7, 51.9	151	44.2	38.9, 49.4	295	45.2
451–749	146	46.9	41.4, 52.5	148	43.3	38.0, 48.6	294	45.0
≥750	21	6.8	3.9, 9.6	43	12.6	9.0, 16.1	64	9.8
Residency								
Study city	73	23.8	19.0, 28.6	61	18.0	13.9, 22.2	134	20.7
Another city in Guangdong province	20	6.5	3.7, 9.3	34	10.0	6.8, 13.2	54	8.4
Another province	214	69.7	64.5, 74.9	244	72.0	67.2, 76.8	458	70.9
STDs related Service								
No	53	21.5	16.3, 26.6	46	17.2	12.7, 21.8	99	19.3
Yes	194	78.5	73.4, 83.7	221	82.8	78.2, 87.3	415	80.7
Syphilis related Knowledge								
No	201	64.6	59.3, 70.0	191	55.8	50.6, 61.1	392	60.0
Yes	110	35.4	30.0, 40.7	151	44.2	38.9, 49.4	261	40.0
Condom use during the last work								
No	64	20.7	16.2, 25.3	69	20.4	16.1, 24.7	133	20.5
Yes	245	79.3	74.7, 83.8	270	79.6	75.3, 83.9	515	79.5
Frequency of condom use in the last month with clients								
Sometimes	109	35.0	29.7, 40.4	130	38.0	32.8, 43.2	239	36.6
Always	202	65.0	59.6, 70.3	212	62.0	55.8, 67.2	414	63.4
Primary sex partners								
No	86	27.7	22.7, 32.7	133	38.9	33.7, 44.1	219	33.5
Yes	225	72.3	67.4, 77.4	209	61.1	55.9, 66.3	434	66.5
Condom use during the last sex with primary partners								
No	120	53.3	46.8, 59.9	70	33.7	27.2, 40.1	190	43.9
Yes	105	46.7	40.1, 53.2	138	66.3	59.9, 72.8	243	56.1
Frequency of condom use in the last month with primary partners								
Sometimes	237	76.2	71.5, 81.0	247	72.2	67.5, 77.0	484	74.1
Always	74	23.8	19.0, 28.6	95	27.8	23.0, 32.6	169	25.9
STDs Symptoms								
No	197	63.3	58.0, 68.7	237	69.3	64.4, 74.2	434	66.5
Yes	114	36.7	31.3, 42.0	105	30.7	25.8, 35.6	219	33.5
Drug Use								
No	307	98.7	97.5, 100.0	339	99.1	98.1, 100.0	646	98.9
Yes	4	1.3	0.0, 2.6	3	0.9	0.0, 1.9	7	1.1
HIV								
No	310	99.7	99.1, 100.0	339	99.1	98.1, 100.0	649	99.4
Yes	1	0.3	0.0, 1.0	3	0.9	0.0, 1.9	4	0.6
Syphilis								
No	291	93.6	90.8, 96.3	329	96.2	94.2, 98.2	620	94.9
Yes	20	6.4	3.7, 9.2	13	3.8	1.8, 5.8	33	5.1
HCV								
No	306	98.4	97.0, 99.8	334	97.7	96.1, 99.3	640	98.0
Yes	5	1.6	0.2, 3.0	8	2.3	0.7, 3.9	13	2.0

**Table 2:** Mean score of each item of GHQ-12 among female sex workers in Guangdong, China.

	Non-depression (*n* = 311)	Depression (*n* = 342)	Total (*n* = 653)
Been able to concentrate on whatever you're doing	0.11	0.11	0.11
Lost much sleep over worry	0.10	0.84	0.49
Felt you were playing a useful part in things	0.09	0.09	0.09
Felt capable of making decisions about things	0.06	0.09	0.08
Felt constantly under strain	0.73	0.93	0.83
Felt you couldn't overcome your difficulties	0.87	0.96	0.91
Been able to enjoy your normal day-to-day activities	0.02	0.06	0.04
Been able to face up to your problems	0.06	0.06	0.06
Been feeling unhappy and depressed	0.59	0.90	0.75
Been losing confidence in yourself	0.86	0.96	0.91
Been thinking yourself as a worthless person	0.85	0.96	0.91
Been feeling reasonable happy, all things considered	0.22	0.19	0.20

**Table 3:** Factors associated with depression among female sex workers in Guangdong, China (*N* = 653).

	Crude model			Adjusted model^*∗*^		
	OR	95% CI	*p* value	OR	95% CI	*p* value
Age (year)						
≤20	Ref					
21–30	0.69	0.43, 1.11	0.124			
31–40	0.87	0.52, 1.45	0.584			
41 and above	0.79	0.46, 1.37	0.407			
Education						
Primary school and below	Ref					
Middle school	0.76	0.52, 1.1	0.145			
Senior high school and above	0.91	0.56, 1.46	0.681			
Marital status						
Unmarried	Ref					
Married	0.84	0.60, 1.17	0.296			
Others	0.78	0.48, 1.25	0.302			
Monthly income						
≤450	Ref					
451–749	0.97	0.70, 1.34	0.837			
≥750	1.95	1.11, 3.45	0.021			
Living status						
Alone	Ref					
With partner	1.20	0.82, 1.75	0.346			
Others	1.14	0.78, 1.66	0.499			
Residency						
Study city	Ref			Ref		
Another city in Guangdong province	2.03	1.06, 3.89	0.032	1.53	0.78, 3.03	0.217
Another province	1.36	0.93, 2.01	0.115			
STDs related service						
No	Ref					
Yes	1.31	0.85, 2.04	0.225			
Syphilis related knowledge						
No	Ref			Ref		
Yes	1.45	1.05, 1.98	0.022	1.45	1.04, 2.03	0.028
Condom use during the last work						
Yes	Ref					
No	0.98	0.67, 1.43	0.910			
Frequency of condom use in the last month with clients						
Always	Ref					
Sometimes	1.13	0.82, 1.57	0.450			
Primary sex partners						
No	Ref			Ref		
Yes	1.67	1.20, 2.32	0.003	1.63	1.14, 2.33	0.008
Condom use during the last sex with primary partners						
Yes	Ref			Ref		
No	0.44	0.30, 0.66	<.0001	0.47	0.31, 0.71	0.000
Frequency of condom use in the last month with primary partners						
Always	Ref					
Sometimes	0.84	0.59, 1.21	0.348			
STDs symptoms						
No	Ref					
Yes	0.77	0.55, 1.06	0.108			
Drug use						
No	Ref					
Yes	0.68	0.15, 3.06	0.615			
HIV						
No	Ref					
Yes	2.74	0.28, 26.42	0.384			
Syphilis						
No	Ref					
Yes	0.58	0.28, 1.18	0.130			
HCV						
No	Ref					
Yes	1.47	0.47, 4.53	0.506			

*Note*. ^*∗*^Adjusted for age, education, marital status, and monthly income.
